# Advances in Wheat Physiology in Response to Drought and the Role of Plant Growth Promoting Rhizobacteria to Trigger Drought Tolerance

**DOI:** 10.3390/microorganisms9040687

**Published:** 2021-03-26

**Authors:** Manon Camaille, Nicolas Fabre, Christophe Clément, Essaid Ait Barka

**Affiliations:** 1 Unité de Recherche Résistance Induite et Bioprotection des Plantes–EA 4707–USC INRAE 1488, Moulin de la Housse–Bâtiment 18, Université de Reims Champagne-Ardenne, BP 1039, CEDEX 2, 51687 Reims, France; manon.camaille@a-r-d.fr (M.C.); christophe.clement@univ-reims.fr (C.C.); 2 Agro-Industrie Recherches et Développements (ARD), Bazancourt Road, 51110 Pomacle, France; nicolas.fabre@a-r-d.fr

**Keywords:** wheat, drought stress, plant growth promoting rhizobacteria, stress tolerance, climate change

## Abstract

In the coming century, climate change and the increasing human population are likely leading agriculture to face multiple challenges. Agricultural production has to increase while preserving natural resources and protecting the environment. Drought is one of the major abiotic problems, which limits the growth and productivity of crops and impacts 1–3% of all land.To cope with unfavorable water-deficit conditions, plants use through sophisticated and complex mechanisms that help to perceive the stress signal and enable optimal crop yield are required. Among crop production, wheat is estimated to feed about one-fifth of humanity, but faces more and more drought stress periods, partially due to climate change. Plant growth promoting rhizobacteria are a promising and interesting way to develop productive and sustainable agriculture despite environmental stress. The current review focuses on drought stress effects on wheat and how plant growth-promoting rhizobacteria trigger drought stress tolerance of wheat by highlighting several mechanisms. These bacteria can lead to better growth and higher yield through the production of phytohormones, osmolytes, antioxidants, volatile compounds, exopolysaccharides and 1-aminocyclopropane-1-carboxylate deaminase. Based on the available literature, we provide a comprehensive review of mechanisms involved in drought resilience and how bacteria may alleviate this constraint

## 1. Introduction

Global agriculture will face multiple challenges over the coming decades, since the demand for food and other plant-based ecosystem services is in constant increase [1]. Further, the world population, totaling around 7.7 billion human beings, is predicted to reach 9.7 billion in 2050 [2]. There is, therefore, an active consideration on appropriate agricultural production practices to reach the goal of higher, but also a sustainable food production to feed world population within the next few decades [3]. In addition, climate change also has a negative impact on crop productivity. Indeed, with climate change, the occurrence of stresses such as drought dramatically increases, becoming one of the most important limiting factors to crop productivity [4]. Further, within the coming epochs, the availability of water for agriculture will be another crucial problem facing the crop production.

During their lives, plants have to cope with many biotic or abiotic stresses, each affecting their development or growth. Among these stresses, biotic stress (caused by bacteria, viruses, fungi, insects, nematodes, etc.) and abiotic stress (such as flooding, cold, heat, salinity or drought) can be distinguished [5]. These constraints are deleterious to crops and subsequently to qualitative and quantitative traits of the yield. Among abiotic stresses, drought is one of the major ones met in recent decades, due to climate change [6]. Particularly, the water deficit affects about two thirds of global land area and about 15% of rural land area [7]. Further, because of increased temperatures, the soil water level is expected to reduce in several areas, leading to the increase the drought in many parts of the world. Consequently, severe morphological, biochemical, physiological and molecular changes might occur in plants [8].

Understanding the impact of the drought on crop production and most importantly, formulating smart strategies to withstand the drought while respecting rules of sustainable agriculture is the challenge for the agriculture of the 21st century. Indeed, to cope with water shortage, crops tolerance might be improved through several methods, including vegetation cover, plant breeding, genetic engineering, more crop lands or farm mechanization. However, most of these solutions are time consuming or cost-intensive, and may even aggravate the climate change and environment problems further. Another solution is irrigation, but global water demand for agriculture will increase by 60% by 2025 [9], which represents more than two thirds of the global consumption of fresh water, meaning that irrigation is not a viable solution [10].

Soil microorganisms with a prospective to alleviate abiotic stress, in addition to plant growth promotion, might be particularly worthwhile tools to ensure a sustainable agriculture [11]. Thus, during the last decades, special attention has been paid to the use of plant growth-promoting rhizobacteria (PGPR) in agriculture to increase productivity and tolerance to both biotic and abiotic stresses as a promising sustainable solution. The knowledge around PGPRs and their modes of action has dramatically increased in the past 20 years [12].

Direct and indirect mechanisms enabling PGPRs to trigger a better plant resilience were identified [13]. Direct mechanisms consist in either the synthesis of plant hormones by these bacteria or the improvement of nutrients assimilation through phosphate or potassium solubilization or nitrogen fixation [14]. On the other hand, indirect modes of action consist in competition with pathogens, synthesizing antibiotics or inducing plant immunity [15].

Reports from the National Science Foundation (NSF) indicate that, in next 30 years, drought stress will adversely impact the yield of crop plants by 400–800 kg/ha [16]. Nowadays, the drought is already estimated to reduce cereal production by 9–10% at a global scale [1]. Among cereals, with around 800 mega tons per year, wheat (*Triticum aestivum*) is one of the world’s most important crop, feeding one-fifth of the world’s human population (source FAO). On a global scale, a reduction of 21% in yields of wheat was recently reported due to drought, thanks to the analysis of data published from 1980 to 2015 [17].

In the current context of both climate change and increasing world population, the main challenge is to increase agricultural productivity, particularly wheat production, in a sustainable and environment-friendly way. This review will, thus, focus on the management of drought stress in wheat crop. The first part will focus on how drought affects wheat physiology and molecular pathways and how wheat plants react to climate changes. In the second part, we will describe the current knowledge of mechanisms allowing PGPRs to improve the tolerance of wheat to drought stress.

## 2. Wheat Drought Interaction: From Perception to Plant Response

The root system is the first plant organ to sense a limitation of water supply and a correlation has been established between improved root system and drought resistance in wheat [18]. Thus, plants are able to perceive a modification of water availability in soil, thanks to a set of specific sensors. However, it remains unclear how these sensors are linked to the responses at the cell and whole plant levels [19].

Water shortage affects all developmental stages by triggering a delay in germination, tillering, booting, heading, anthesis, grain filling and maturity [20]. The flowering and the grain filling periods seem to be the most susceptible periods [21]. Therefore, a drought stress occurring at one of these two developmental stages is called terminal drought and leads to substantial crop yield losses, depending on the severity and duration of the stress [20,22]. Regarding flowering, the female meiosis is the most critical period. At this stage, the drought inhibits the completion of meiosis in the ovule, leading to abnormal female reproductive cell, thus limiting the success of reproduction [23,24].

This section will review physiological and molecular pathways affected by the drought stress. Then, the mechanisms deployed by wheat plants to mitigate the negative effects of drought stress on these pathways will be examined.

### 2.1. Water Balance and Osmotic Adjustment

The drought stress affects wheat plants by decreasing water content and turgor of plant cells and tissues and a strong positive correlation was established between leaf water potential and photosynthetic rate [25,26,27]. Decreased water content in plants leads to a higher concentration in all cellular components and to an increase in viscosity of the cellular content, which can be toxic and harmful for the enzyme’s activity [4]. The water flux from xylem to cells in elongation is reduced, thus decreasing turgor and inhibiting the cell elongation [7]. Furthermore, the mitosis is also inhibited, reducing the cell division [4]. Taken together, these two parameters lead to a global inhibition of the plant growth, with a decrease of yield as a consequence [28].

Plants are able to limit water losses by modifying their osmotic potential. Indeed, as the turgor decreases, the plant must react by decreasing its water potential and thus maintaining osmolarity and pH to sustain life and to avoid dehydration. In this regard, osmotic adjustment (OA) is allowed by the production and accumulation of soluble molecules, called osmolytes, such as proline, glycine betaine, polyamines, polyols, soluble sugars and ions (potassium for example) by plant cells. Stress-induced accumulation of these metabolites lowers the water potential of the cell, promoting water retention in the plant without interfering with a normal metabolism [22]. Therefore, in wheat, a positive correlation between OA and grain yield was shown [29]. Sugars and proline may help to stabilize proteins and cell structures, particularly when the stress becomes severe or persists for longer periods.

#### 2.1.1. Sugars

The concentration of soluble sugars and starch in plants is affected by both environmental conditions and varietal characteristics. In wheat, soluble sugars make the largest contribution to OA when subjected to the drought stress. The total soluble sugars content may increase by 80% in wheat seven days after exposure to the drought stress [30,31]. Among soluble sugars, glucose represents the main one (about 85%) involved in OA [32]. Starch is also emerging as a key molecule in modulating plant responses to water deficit. Degradation of starch in the light by stress-activated β-amylase1 to release sugars and sugar-derived osmolytes has been often associated with enhanced tolerance [33].

#### 2.1.2. Proline and Other Amino Acids

The proline concentration may increase by 90% in wheat after a 7 day drought stress [31]. For instance, the proline level increases from about 1 µmol·g^−1^ of leaf dry weight (DW), when wheat is well-watered, to 11 µmol·g^−1^ of leaf DW after 15 days without water [34]. The observed increase in free proline may be attributed, in part, to enhanced expression of genes involved in proline biosynthesis and contributes to OA and protection of the subcellular components [35,36]. However, the accumulation of proline slightly contributes to OA but is mainly involved in the protection of organs and cellular functions [30]. Accumulation of glycine betaine during drought stress could alleviate effects of drought stress on photosynthesis through the improvement of OA [37,38]. The cell concentration in other amino acids also increases during a drought stress, due to the degradation of proteins, which is a reaction contributing to OA, but also due to a de novo synthesis of amino acids [30,39].

### 2.2. Photosynthesis and Gaseous Exchanges

**Figure 1:**
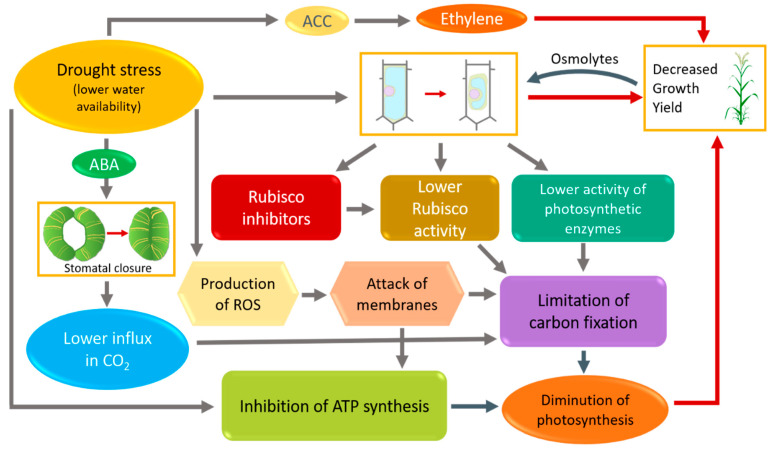
Impact of drought stress on plant photosynthesis, growth and yield (inspired from Farooq et al., 2009). ABA: abscisic acid; ACC: 1-aminocyclopropane-1-carboxylate; ROS: reactive oxygen species.

Ninety percent of crop biomass is derived from photosynthetic products. Therefore, the photosynthesis process is vital for plant and a highly positive correlation was shown between potential leaf photosynthesis and maximal crop growth and yield [40]. Photosynthesis is the physiological trait that is the fastest affected under drought stress conditions (Figure 1). The water stress at grain filling stage triggers a decrease of photosynthetic activity and hastened the leaf senescence, resulting in abbreviated grain filling period [41,42]. The related impact of drought stress on reducing the grain filling is due to the lower performance of photosynthesis and carbon assimilation [22]. The dysfunction of photosynthesis originates either from the limited access to atmospheric carbon dioxide through stomatal closure or to the alteration of the photosynthetic apparatus [28,43,44]. One of the first effects of drought stress is the limitation in CO_2_ influx, thus decreasing the carbon assimilation by the photosynthetic apparatus [45]. This is mainly due to the stomatal closure as a result of convergent parameters such as a limitation of water content in guard cells, a lower external humidity or the synthesis of drought related phytohormones, including abscisic acid (ABA) [4].

Different steps of the CO_2_ diffusion are affected under low water supply regime, i.e., the limitation of stomatal conductance (g_s_) and/or mesophyll conductance to CO_2_ (g_m_) [46,47]. A photoinhibition also occurs when the cell cannot dissipate the excess of the light energy [7]. The photoinhibition is defined as the decrease in photochemical efficiency experienced in response to intense illumination due to radiation damages [48]. Usually, the light energy is transformed in electron and then produces energy (ATP and NADPH) used for the fixation of CO_2_ in the Calvin cycle. When the plant faces drought stress, the carbon fixation is limited and the rate of absorbed light exceeds the rate of light used in the chloroplasts, which, finally, leads to an enhancement of the photoinhibition [49]. Moreover, drought stress triggers the interruption in the protein synthesis and their misfolding, including photosynthetic enzymes [7,50]. In particular, it has been reported in wheat that water shortage directly impacts the Rubisco efficiency by decreasing its content and activity [22]. Rubisco activity may also be decreased by inhibitors during drought stress [51]. The ATP synthesis is also impaired by drought stress because of the down-regulated electron transport and membrane’s damages [22]. The collection of light energy is altered under drought stress. It has been shown that chlorophyll is photo-oxidized under low water conditions [52,53]. Photooxidative stress is mainly due to an excessive absorption of light excitation energy leading to over-reduction of the electron transport chains, therefore generating reactive oxygen species [54,55].

### 2.3. Oxidative Status

Under drought stress, the reactive oxygen species (ROS) production is increased in various ways. The limitation of CO_2_ fixation will decrease NADP^+^ regeneration during the Calvin cycle, therefore triggering a decline of the photosynthetic electron transport chain. Indeed, there is a greater leakage of electrons to O_2_ by the Mehler reaction through the photosynthesis under drought stress, reaching 50% in drought-stressed wheat [56]. In addition, drought stress induces an oxidative burst, which causes many damages to plant cells leading, without plant response, to death [57]. Chronologically, there is first an increase in ROS content in plant cells, then an increase in expression of genes encoding antioxidants and, finally, an intensification in antioxidative systems leading to a better drought stress tolerance [58].

#### 2.3.1. Reactive Oxygen Species (ROS)

Taken together, the exposition of plant to permanent excessive light and the reduced CO_2_ intake bring the electrons towards oxygen molecule, leading to the production of ROS [59]. Indeed, when the light harvested by the photosystems cannot be used for photosynthesis or photorespiration anymore and cannot be dissipated into heat, it triggers an oxidative burst, a mechanism common to many stresses [60]. Among the plant cell components, membrane lipids and macromolecules such as DNA and proteins may be damaged by ROS [61], such as hydrogen peroxide (H_2_O_2_), superoxide radical (O_2_^●−^), hydroxyl radical (HO^●^) or singlet (^1^O_2_).

#### 2.3.2. Antioxidant Systems

To cope with oxidative stress, wheat plants produce several antioxidants, including enzymatic and non-enzymatic ones [22]. Among the antioxidant enzymes, the best described are catalase (CAT), superoxide dismutase (SOD), glutathione peroxidase (GPx), ascorbate peroxidase (APX) and glutathione reductase (GR) [2,5]. These enzymes are involved in the degradation of the ROS and, thus, in the maintenance of the plant cells vital functions. Their expression and activity are often enhanced in wheat during drought stress, depending on the stress intensity and duration, as well as developmental stage [2,61,62,63]. For instance, the activity of the two main enzymes of the ascorbate/glutathione scavenging pathway, i.e., APX and GR activities were increased in response to drought stress [64,65]. Apart from enzymes, plant cells also produce non-enzymatic antioxidants, such as ascorbate or glutathione, which similarly contribute to ROS scavenging or avoidance under drought stress. Higher glutathione and ascorbate amounts were reported in wheat subjected to drought, which were associated with a better tolerance to drought stress [66]. Wheat plants can also produce other antioxidant molecules, such as α-tocopherol, carotenoids or glycine betaine [38,67]. Accumulation of α-tocopherol, a potent protector of thylakoids and chloroplasts membranes, has been reported in several plant species under the drought stress [65,68].

### 2.4. Hormonal Balance

**Figure 2:**
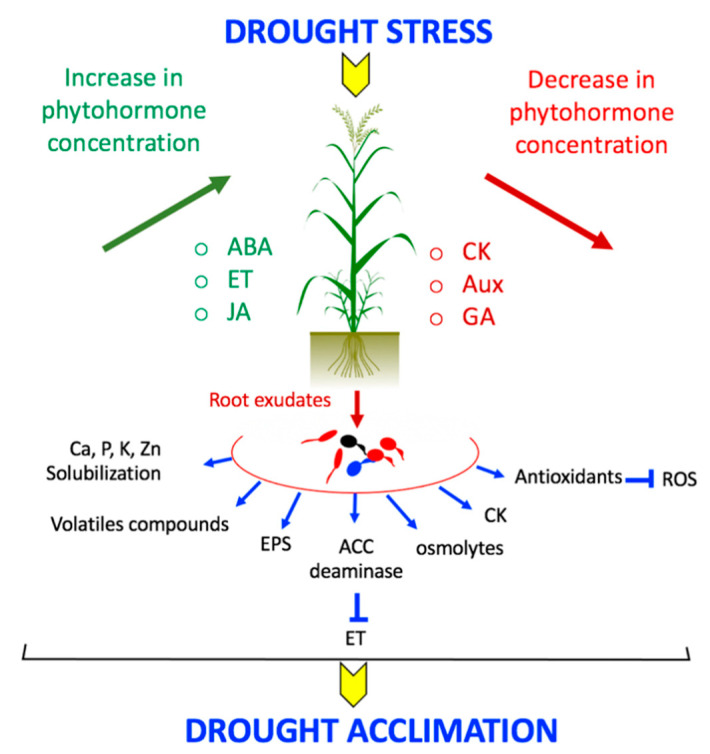
Hormonal changes in wheat during drought stress with the impact of rhizosphere bacteria. ABA: abscisic acid; EPS: exopolysaccharides; ET: ethylene; JA: jasmonic acid; CK: cytokinin; Aux: auxin; GA: gibberellin, ROS: reactive oxygen species.

As observed for many plant species, wheat plants undergo considerable hormonal changes and modulation of the hormonal balance during a water shortage (Figure 2) [69]. Plant phytohormones are crucial for the capacity of plants to adapt to a situation, through modulation of growth, development, nutrient allocation and source/sink transition [70]. A lot of them are involved in the plant reaction to drought stress, such as abscisic acid (ABA), cytokinin (CK), ethylene, auxin (IAA), salicylic acid (SA), brassinosteroids (BR) and jasmonic acid (JA) [70].

One of the pivotal events in osmotic stress responses is the fast, transient accumulation of abscisic acid, which facilitates stomatal closure and expression of ABA responsive genes that protect plant from further water loss and damage [22,36]. The production of ABA is one of the early hormonal responses to drought stress [70]. Most often, ABA is synthesized in the dehydrated roots facing a dry soil, then transported to leaves through the xylem allowing a long-distance signaling of water deficit in the plant [71]. In wheat, the amount of ABA is often negatively correlated to photosynthesis efficiency and chlorophyll content in the flag leaf and seems to contribute to pollen sterility [22]. In fact, the regulation of endogenous ABA level is important for pollen development and high levels of ABA could lead to pollen sterility in wheat [72]. Several transcriptomic analyses have reported an ABA-dependent induction of a multitude of dehydration-stress related genes, such as transpiration minimizing genes or oxidative stress related genes, as well as some of those involved in the primary carbohydrate metabolism [73,74,75,76]. In addition, the ABA-responsive genes encode enzymes or proteins involved in tolerance to drought stress (see “Specific proteins” section). In line with transcriptomic and metabolomic data, Thalmann et al. [33] conclude that de novo biosynthesis of ABA triggers starch degradation in the light in response to osmotic/dehydration stress.

Cytokinins are the antagonist of ABA since they stimulate the stomatal opening and reduce the sensitivity of the stomata to ABA by delaying senescence [60]. In wheat, CKs concentration is often positively correlated to photosynthesis level and chlorophyll concentration within the flag leaf [22]. In transgenic plants overexpressing cytokinin, the plant senescence triggered by stresses like drought was delayed in the presence of high levels of cytokinin [77,78]. In response to drought, these plants remain green and healthy while non-transformed plants are totally wilted. The enhanced amount of CKs increases sink strength by over-expressing genes involved in cell division through sugar signaling, which involves increased phloem unloading but also the sugar import to endospermic cells via the cell wall-associated enzyme invertase [79]. A treatment with CKs allows higher biomass and yield in drought-stressed plants, through an improvement of N metabolism, but the CK concentration often decreases during a drought stress [80]. Increasing evidence has reported that CKs regulate plant drought acclimation/adaptation across a multistep phosphorelay pathway [81]. Nevertheless, several studies highlighted the multidimensional nature of cytokinins as they can have both positive and negative impacts on drought tolerance [82,83].

Present in all higher plants, ethylene is involved in a multitude of plant processes including for example, growth of roots, leaves, flowers, fruits, rhizobia nodulation of legumes and plant-mycorrhizal fungi interactions [3]. The amount of ethylene produced by the plant increases dramatically during a drought stress until it reaches a threshold, about 25 g·L^−1^ [84,85,86]. Once this level is reached, the ethylene called “stress ethylene” becomes harmful for root and shoot growth and leads to senescence, chlorosis and leaf abscission [3,86]. Yang et al. [41] found that during severe drought stress, the concentration of ethylene (and its precursor ACC) increased by about 2 times compared to the non-stressed control, while the grain-filling rate and the final grain weight significantly decreased. Ethylene can also induce the expression of drought-specific genes, through the ethylene responsive element binding proteins (ERF), which are transcription factors involved in the response to water stress (see “Specific proteins” section) [87].

The best-known functions of auxins are their role in the rhizogenesis through the initiation of lateral and adventitious roots and the stimulation of cell division [28,88]. Interaction between auxins and ethylene modulate the root development and architecture and, therefore, it is considered as a key aspect of drought tolerance [89]. In fact, genes controlling root system architecture (RSA) have been the target for molecular breeding to improve the plant drought tolerance [90]. It was shown that an improved root system is associated with a better drought tolerance in wheat [18]. Thus, auxins have an indirect but important role for the plant tolerance to drought stress. Auxins are able also to modulate root hydraulic properties by enabling the expression of water-saving traits, associated with enhanced yields under drought stress [91]. However, drought stress seems to reduce the auxin biosynthesis and signaling pathway in wheat. Indeed, the plant auxin content decreased by more than 30% whereas an upregulation by more than 2 folds of the AUX/IAA1 gene, encoding transcriptional repressor of auxin-responsive genes, was observed [45].

Brassinosteroids (BR) were reported to induce the expression of stress responsive genes, leading to the conservation of the photosynthetic activity, activation of antioxidative enzymes such as SOD, peroxidase and catalase, accumulation of osmolytes like proline and soluble sugars and induction of other hormonal responses, which could help plant to withstand the drought stress [70,92,93].

### 2.5. Transcriptional Regulatory Network

**Figure 3:**
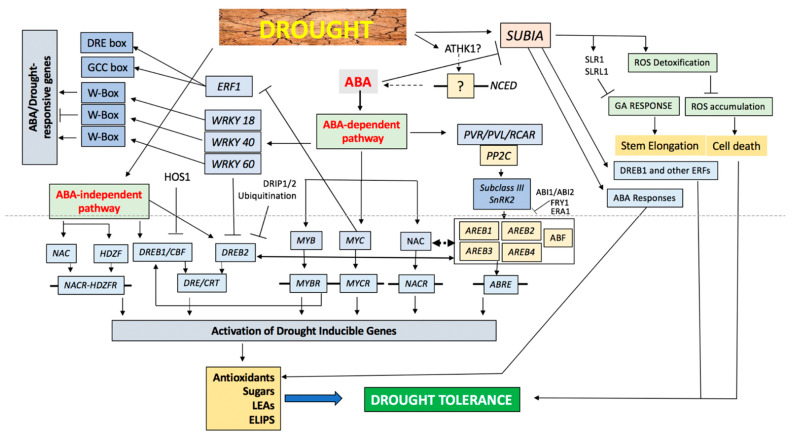
A schematic model of transcriptional regulatory networks and gene expression in the drought stress signals.

At molecular level, the drought adaptative mechanisms include the regulation of gene expression and the identification of transcription factors. Deciphering mechanisms by which these elements are modulated under drought stress and the various triggered responses would be decisive to induce plants’ stress tolerance (Figure 3).

As stated earlier, ABA plays a central role in drought regulation by addressing the water deficit and modulating the stress response by controlling stomatal movement and triggering appropriate genes. Several genes involved in the biosynthesis of ABA are intensely triggered under drought stress, including genes encoding NCED (9-cis-epoxycarotenoid dioxygenase), a key enzyme of ABA biosynthesis. While some genes are triggered by ABA application, a large number of drought-inducible genes are not induced by ABA treatment, signifying the existence of another ABA-independent pathway in the drought adaptative response [94]. Both ABA-dependent and ABA-independent pathways modulate the transcriptional response by disturbing one or more regulons active under drought stress [95].

Drought activates many pathways in plants that have been broadly classified in two categories, i.e., ABA-dependent pathways and ABA-independent pathways. These stress-inducible transcription factors involve members of the DRE-binding protein (DREB) family, the zinc-finger family, the ethylene-responsive element binding factor (ERF) family, the MYB family, the basic helix–loop–helix (bHLH) family, the basic-domain leucine zipper (bZIP) family, the NAC family, the WRKY family and the homeodomain transcription factor family. These transcription factors modulate diverse drought inducible genes and constitute gene networks.

#### 2.5.1. ABA-Dependent Network

The MYC/MYB transcription factors play a significant role in drought stress signaling as they have been induced under drought stress [96]. The synthesis of MYC and MYB proteins after the accumulation of endogenous ABA demonstrate their role is in a late stage of the stress responses. The NAC (NAM, ATAF1 and CUC2) transcription factors are also one of the biggest transcription factors families in plants that act in response to various environmental stresses, including drought [97]. Several NACs have been reported to be highly activated under drought [98] and regulate gene expression in ABA-independent manner.

In ABA-responsive gene expression, the ABRE (ABA-responsive element) is a major cis-acting element. In ABA-deficient aba2 mutants and in ABA-insensitive abi1 mutants, the AREB/ABF proteins have a reduced activity demonstrating the role of ABRE in ABA-mediated signal. In addition, transgenic plants overexpressing AREB1/ABF2, AREB2/ABF4 or ABF3 have an enhanced drought tolerance and a better sensitivity to ABA. These results highlight the central role of these transcription factors that cooperatively function in ABA-dependent transcriptional activation through their ABREs under drought stress conditions [94]. Nevertheless, Singh and Laxmi [99] reported that AREB/ABFs induced DREB2A and AREB/ABFs interact with DREB2A, suggesting that a crosstalk between ABA-dependent and ABA-independent pathways exists under drought stress.

ABA signal perception also induces WRKY18 and WRKY40 and their products might bind to W-box present in WRKY60 and, thus, trigger it. In response to drought stress, these three WRKYs are exported from nuclei and interact with magnesium-protoporphyrin IX chelatase H subunit in the chloroplast to mitigate the negative impact of the drought stress-inducible target genes [100].

During drought stress, another transcription factor that goes to the bZIP transcription factor subfamily is ABA-responsive element-binding proteins/factors (AREBs/ABFs) that are ABA-dependent and upregulated.

The PP2C is a negative regulator of ABA signaling that dephosphorylates and, thus, inactivates the subclass III SNF1-related protein kinases 2 (SnRK2s), which in turn phosphorylate AREB/ABF. As central components in ABA signaling, the three subclass III SnRK2s participate in the convergence of ABA-dependent and ABA-independent pathways, regulating the expression of AREB/ABFs and DREB under drought stress and consequently trigger the expression of AREB/ABF regulon genes. Further, the ABA-activated SnRK2 protein kinase participates controlling stomatal closure [101].

#### 2.5.2. ABA-Independent Network

ABA-independent networks are specially enhanced in response to jasmonic acid (JA), gibberellin and salicylic acid stimuli. The modulation of these ABA-independent genes occurs through the DRE and CRT cis-acting elements, in combination with DREB or CBF transcription factors [102]. DREB1/CBF and DREB2 both belong to plant-specific AP2 (APETALA2)/ERF (ethylene-responsive element-binding factor) family, possessing an AP2/ERF DNA-binding motif. Morimoto et al. [103] have reported that stabilization of DREB2A is essential but not sufficient to trigger downstream genes suggesting that, in addition to DREB2A-interacting protein 1 (DRIPs), other factors might be implicated to activate or degrade the DREB2A.

MYB/MYC and WRKY are other transcription factors, which have been demonstrated to be involved in regulating the response to drought stress in plants trough ABA-independent signaling manner [104]. Similarly, in addition to their role in the ABA-dependent signaling network, NAC transcription factors are also playing an important role in the ABA-independent signaling pathway [105]. The gene ANAC096, which encodes NAC transcription factor in the ABA-independent signaling pathway, interacts physically with the ABA-dependent transcription factors ABF2 and ABF4 to modulate gene expression under drought stress [106].

### 2.6. Specific Proteins

Drought stress induces the expression of drought-responsive genes, leading to the production of specific proteins. The regulatory proteins, which are transcription factors such as kinases, phosphatases or calmodulin-binding proteins, were discriminated from functional proteins. In the latter group, the proteins directly act to help plant to cope with the drought stress. These proteins include chaperones, late embryogenesis-abundant (LEA) proteins, enzymes for osmolytes biosynthesis and water channel proteins [39].

Dehydrin (DHN) genes belong to the LEA family and are up regulated in wheat in response to stress such as drought, leading to cell dehydration. In wheat, they encode DHN proteins, which have a role in protection mechanisms [107]. The expression of many DHNs is induced by ABA; thus, they are also referred as RAB proteins (responsive to ABA). DHN proteins may bind to the partly dehydrated surface of proteins, protecting them from protein denaturation. They may also exhibit ROS scavenging properties [108].

The dehydration responsive element binding proteins (DREB) genes are specifically induced by drought stress and encode transcription factors, which belong to the ERF protein family (Figure 3, “ABA-independent pathway”). These transcription factors trigger the expression of abiotic stress-responsive genes, such as aquaporin genes, conferring, therefore, a certain level of tolerance to the plant [109,110].

ABA is able to induce the production of other specific proteins, such as aquaporins or Acetyl-CoA Carboxylase, a key enzyme in lipid metabolism [60,75]. There are two types of ABA-dependent pathways (Figure 3): the first requires new protein (transcription factor) synthesis, while the second does not. In the latter, the promoter domain has an ABA-responsive element ABRE (ABA-Responsive Element), with a very specific sequence. Gene expression is induced when the corresponding transcription factor binds to ABRE after being modified (for example, phosphorylated) by an ABA-activated protein (for example, a protein kinase). The transcription factor belongs to the bZIP (basic leucine zipper) family. In the route where a new protein synthesis is required, a Myc transcription factor bind to the ABA-responsive element (which is not an ABRE). In this case, the synthesis of the Myc factors is required before the induction of the ABA-responsive genes [71].

Heat Shock Proteins (HSPs) form a large protein family including proteins of different physiological functions and are known to be involved in the plant abiotic stress response. HSPs are chaperones which prevent protein misfolding and maintain proteins in their functional conformation [39]. These proteins are produced in response to a heat stress but also to a wide range of other stresses, such as drought. There are several types of HSPs, which are complementary and altogether maintain the plant cell homeostasis. For example, Hsp70 chaperones interact with a wide range of co-chaperones proteins and assist protein-folding processes, while Hsp100 chaperones participate to protein disaggregation and/or degradation, by removing misfolded or denatured proteins that may thus, be harmful for the cell [111]. In wheat, drought stress results in increasing the expression of the gene encoding HSP17.8 up to 3-fold, when compared to non-stressed control [61].

### 2.7. Production of Volatile Organic Compounds

VOCs (Volatile Organic Compounds) are volatile molecules of low molecular weight produced by plant leaves and their production is known to be induced by several stresses, including drought stress. These molecules are used as a signal to communicate within the plant and with other plants and trigger a stress tolerance to these plants [52]. Among plant emitted VOCs, isoprenoids dominate the emissions and in particular, isoprene. This VOC confers the stress tolerance through different mechanisms, including the stabilization of the chloroplastic (thylakoid) membranes. Isoprene is able to occupy the space between the lipid tails, increasing the adhesive forces and acting as molecular glue [112]. Another way for isoprene to increase plant stress tolerance is to act as antioxidant. In fact, it was shown that the presence of isoprene maintains ROS and the level of lipid peroxidation much lower than without [112]. It has been shown that the emission of stress-specific-VOCs is increased under drought stress in wheat, proportionally to the severity of the applied stress, highlighting the crucial physiological and ecological roles of stress-released VOCs when plants are subjected to stress [63]. However, the production of VOCs has a cost in terms of carbon, since the more VOCs are emitted, the less carbon is fixed by photosynthesis, which might lead to reduced plant growth in non-stressed circumstances.

### 2.8. Lipids and Cell Membrane Stability

Cell membrane stability is one of the sub-traits that has been used to estimate the impact of drought and, therefore, to screen tolerant genotypes [113]. The cell membranes damages are indeed the earliest event in plant exposed to drought stress, due to oxidative stress resulting from ROS, which leads to lipid peroxidation and, consequently, membrane injuries, enzyme inactivation and protein degradation. It is generally accepted that the maintenance of cell membranes’ integrity and stability is a key factor in drought tolerance [114]. Therefore, the cell membrane stability and reciprocal cell membrane injuries are physiological markers to evaluate the plant’s drought tolerance [28,115]. Some mechanisms previously described might help plants to limit damages to cell membranes. For example, tocopherols, which are antioxidants were shown to protect lipids and other membranes. In addition, production of osmolytes, such as proline or glycine betaine, stabilize membranes, as well as LEA proteins, which protect lipid membranes [57]. In addition, polyamines might be linked to membrane’s anionic components, including phospholipids, protecting the lipid bilayer from deleterious impacts of stress [116].

## 3. Wheat Drought Acclimation by PGPRs

**Figure 4:**
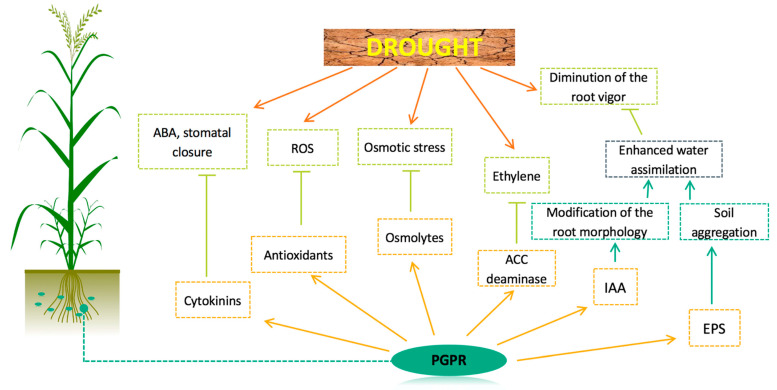
Wheat drought acclimation by PGPRs (inspired from Kaushal and Wani, 2016b). ROS: reactive oxygen species; ACC: 1-aminocyclopropane-1-carboxylate; IAA: indole acetic acid; EPS: exopolysaccharide.

Despite the several mechanisms developed by wheat plants to cope with drought stress, significant losses are generally observed when wheat plants are exposed to severe water limitations. In this context, the action of some PGPRs may help to further restrain the negative effects of drought stress. Indeed, PGPRs may have beneficial effects on plants as they enhance nutritional capacity and increase resistance to both biotic and abiotic stresses, including drought and pathogen infection [117,118]. In this context, PGPRs can be useful allies for plants, as they have several modes of action to improve tolerance to drought stress (Figure 4).

### 3.1. Physiological Effects of PGPRs on Plant under Drought Stress

PGPRs may positively impact the health of wheat exposed to drought stress through their action on different physiological processes. First, PGPRs are able to improve growth and yield of wheat plants. For example, the inoculation of 1-month old wheat plants with either strain *Bacillus safensis* W10 or *Ochrobactrum pseudogregnonense* IP8, lead to higher root and shoot dry weight in six varieties of wheat subjected to drought stress [2]. In addition, the inoculation of wheat with *Azospirillum lipoferum* B3 increased the final yield after a drought stress during flowering, when compared to non-inoculated plants [119]. More recently, Chen et al. [120] have reported that *Pantoea alhagi* triggers an enhanced growth and drought tolerance in wheat.

PGPRs are able to modify the RSA and the structure of root tissues [121]. Inoculation of wheat seedlings with PGPR strains increased root elongation and root dry weight under water shortage, compared to non-inoculated plants [122]. Water stressed wheat plants inoculated with strain *Azospirillum* sp. B3 also showed a better root growth, leading to increase nutrient and water assimilation, due to production of phytohormones by this bacterium [119]. Strains *Bacillus amyloliquefaciens* 5113 and *Azospirillum brasilense* NO40 also improve drought stress tolerance in wheat, likely acting by increasing root growth and lateral root formation [61].

The plant colonization with beneficial bacteria probably triggers several mechanisms to help plants to withstand photosynthesis under stress conditions. For example, inoculation of wheat with strain *Bacillus thuringiensis* AZP2 lead to much higher net assimilation rate under drought, when compared to non-primed plants and it was correlated with higher survival rate [63]. Wheat inoculated with strain *Burkholderia phytofirmans* PsJN exhibited higher photosynthetic rate and chlorophyll amounts related with a higher grain yield, when compared to non-bacterized plants [123]. Inoculation of wheat with beneficial PGPRs improves maximum photosynthetic efficiency of photosystem II (F_v_/F_m_), net CO_2_ assimilation, stomatal conductance and transpiration rate under drought conditions. The enhanced photosynthesis leads to more biomass, measured as shoot and root dry weights and length [45].

In plants, electrolyte leakage (EL) and malondialdehyde (MDA) contents, a product of lipid peroxidation, are reliable indicators of oxidative membrane damages due to stress. Avoiding damages caused to cell membranes is a key point for plant to resist to drought stress. Some PGPRs are able to help plant in this regard. Inoculation of wheat seedlings with the strain *Klebsiella* sp. IG 3 leads to much lower EL and MDA under drought conditions when compared to non-inoculated plants [31].

Benefices of PGPR on plants in drought conditions are based on different mechanisms, which will be presented in the next paragraphs, following the actual state of the art concerning these mechanisms.

### 3.2. Osmolytes Production/Modification of the Water Status

The OA is one of the mechanisms used by plants to cope with drought stress. Indeed, in response to drought, plants modulate their tissues turgor by adjusting OA and thus, maintain the cell homeostasis. Some PGPRs are able to produce osmolytes, which would act in synergy with those produced by the plant and may favor the plant tolerance to drought or salt stress [52]. These compatible solutes can be sugars, quaternary ammonium compounds, polyhydric alcohols, proline and other amino acids, or water stress proteins such as dehydrins. Moreover, several bacteria are able to alleviate the water stress in plants by triggering the production of osmoprotectants in their host plants [52].

Many studies on PGPR have been performed measuring relative water content (RWC) in water stressed wheat plants inoculated or not with beneficial microorganisms [38,107,115]. In wheat, inoculation with the PGPR strain *A. brasilense* Sp245 may confer a better OA and water status, triggering yield increased by 17% [124]. In addition, wheat plants inoculated with either strain *Bacillus safensis* W10 or *Ochrobactrum pseudogregnonense* IP8 exhibited an increase of proline concentration in their leaves and their RWC under drought stress conditions [2]. Unfortunately, the experiment did not allow to link directly bacterial osmolytes production and the increase of proline concentration in wheat plants. These improved parameters were associated with an increased activity of antioxidant enzymes, such as SOD, CAT or GR, and lead to higher root and shoot dry weight in wheat [2].

In some cases, a decrease in osmolytes content in plant exposed to drought stress has been reported. For instance, an inoculation of wheat seedlings with the strain *Klebsiella* sp. IG 3 leads to a significant decrease in total soluble sugars and proline contents under drought conditions, when compared to non-inoculated plants. However, inoculated plants exhibit higher root length and number, enhanced fresh and dry weight of shoots and roots under the same conditions. In such a case, it is likely that the selected PGPR has another mode of action, such as the production of biofilm, which reduces the stress upstream [31].

### 3.3. Modification of the Antioxidant’s Activity/Concentration

The antioxidant enzymes activity and related molecules are increased under drought stress in plants. The inoculation of PGPR may improve this adaptive process, helping plants to face water shortage. In seedlings subjected to water shortage, inoculation with the strain *Bacillus thuringiensis* AZP2 enhanced the activity of the ROS-scavenging enzymes, such as GR, SOD and CAT, leading to better survival of the seedlings [63].

In addition, when applied on wheat seeds, strains *Bacillus safensis* W10 or *Ochrobactrum pseudogregnonense*, IP8 improved the activity of antioxidative enzymes and triggered the accumulation of non-enzymatic antioxidants under drought conditions [2]. Here, again, the activity of peroxidase (POX), CAT, ascorbate peroxidase (APX), SOD and GR was enhanced, whereas the concentrations of carotenoids, ascorbate and proline were also increased during the stress. The modification of these parameters diminished the oxidative stress and lead to a more antioxidative status, resulting in higher root and shoot wheat biomass. In these studies, we do not really know if the bacteria triggered an increased production of antioxidants in the plant or if the increased antioxidant activity and amount was due to the bacterial production of antioxidants.

Nevertheless, in some cases, a decreased activity of wheat antioxidative enzymes was reported after PGPR inoculation. For example, in water-stressed wheat seedlings, the inoculation of strains *Bacillus amyloliquefaciens* 5113 and *Azospirillum brasilense* NO40 leads to a weaker activity of ascorbate peroxidase and dehydroascorbate reductase (DHAR), when compared to non-primed plants. Nevertheless, bacterial priming leads to less oxidative stress, to better survival rate and to higher growth parameters, such as fresh and dry weights and water content [61]. In this case, it is likely that the selected PGPR has another mode of action, such as the production of ACCd or IAA, leading to the reduction of the stress upstream [31].

### 3.4. Production of Exopolysaccharides (EPS)/Biofilm

Exopolysaccharides (EPS) are polymers produced and secreted by some bacteria. EPS are one of the main components of bacterial extracellular matrix, which often contribute to 40–95% of the bacterial weight [125]. EPS are a complex mixture of biomolecules such as proteins, humic-like substances, polysaccharides, neutral sugars, uronic acids, amino sugars, organic ester-linked substituents and pyruvate ketals, nucleic acid, lipids and glycoproteins. The production and composition of EPS depend on the bacterial growth phase, the medium composition and the environmental conditions [125]. They have several interesting properties, including the protection of the bacteria against desiccation [126] and a huge water retention capacity of up to 70 g water per g polysaccharide [125].

EPS production is of great interest and is often used as an in vitro parameter for PGPR screening or characterization. When EPS-producing PGPRs are in the plant rhizosphere, they lead to a better soil aggregation around the roots and more efficient water and nutrient flux toward the plant roots [126,127]. Further, bacteria-produced EPS enhance the root adhering soil (RAS) permeability and may form a protecting biofilm [126]. Most of these properties make the EPS-producing PGPRs promising for the amelioration of the wheat plant tolerance to water stress. For example, some bacteria belonging to the genus *Pseudomonas* have the intrinsic ability to face drought stress conditions by producing EPS [128]. The strain *B.thuringiensis* AZP2 produces high quantity of biofilm on the roots when inoculated on wheat. Two to three times more soil was aggregated around wheat roots under water stress, compared to non-inoculated plants, allowing an increase in water use efficiency by 63% in inoculated wheat. This was associated with a higher survival rate of wheat to drought stress [63]. In the same line, the inoculation of wheat seedlings with EPS-producing strain *Klebsiella* sp. IG3 led to improved RAS permeability through increasing soil aggregation and water potential around the roots [31].

Bacterial EPS secretion of the strain *Pantoea agglomerans* NAS206 increased the root adhering soil/root tissue ratio, which allowed a better aggregation of the soil surrounding the wheat roots [129]. Thus, it improved the RAS permeability and maintained a better water potential around the roots, thus allowing a better assimilation of water and nutrients uptake by the plant [31,129].

### 3.5. Production of Volatile Organic Compounds

Volatile organic compounds are usually produced by plants but may also be produced by some PGPRs [52]. PGPRs may produce their own VOCs, such as 2,3-butanediol, acetoin or acetic acid [130]. The function of these bacterial VOCs seems to be slightly different from those produced by plants by acting as signaling molecules to mediate plant-microorganism interactions [117]. For example, bacterial acetic acid enhances the formation of biofilm formed by EPS produced by certain PGPRs. The 2,3-butanediol seems to induce the plant drought stress tolerance through stomatal closure and reduced water loss [130]. Genes involved in this pathway, including budA, budB and budC were characterized in the biocontrol agents *Klebsiella terrigena* and *Enterobacter gerogenes* [131].

In some cases, the inoculation of wheat with PGPRs may reduce the emission of VOCs and, thus, maintain the level of photosynthesis. In wheat plants primed with strain *Bacillus thuringiensis* AZP2, the emissions of VOCs were lower [63]. The emission of β-pinene and benzaldehyde, which are terpenoid and benzenoid VOCs, increased in non-primed plants subjected to drought stress but the inoculation of strain *B. thuringiensis* AZP2 resulted in the diminution of VOCs emissions by half. The emission rate of geranyl acetone was maintained to its basal level in primed-wheat plants, at the same level as non-stressed plant. There was a strong negative correlation between the emission of these VOCs in primed-plants and the survival rate and even the net photosynthesis rate under water stress conditions [63].

### 3.6. Production of ACCd

**Figure 5:**
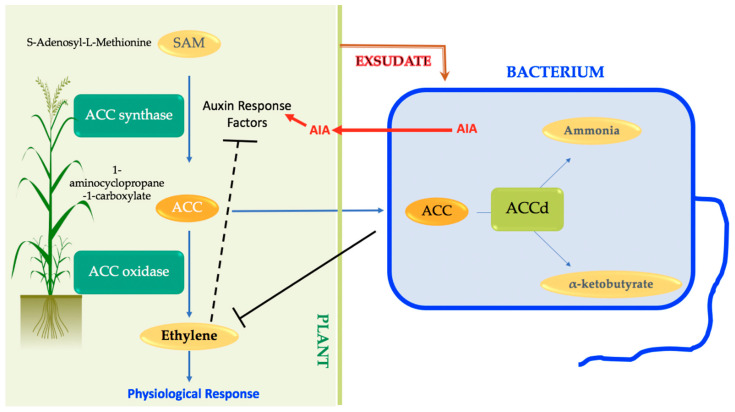
Model for how the ACC deaminase lowering of ethylene levels modulate physiological response.

Some PGPRs are able to produce the 1-aminocyclopropane-1-carboxylate deaminase (ACCd) [132,133], which degrades ACC, the direct precursor of ethylene [134]. The bacterial ACCd degrades the ACC into ammonium and α-ketobutyrate and, thus, reduces the amount of plant ethylene (Figure 5; [85]). The ACCd is found in a wide range of PGPR genus, such as *Pseudomonas*, *Bacillus*, *Rhizobium*, *Sinorhizobium*, *Variovorax*, *Burkholderia* or *Azospirillum* [134,135,136]. PGPRs containing ACC deaminase increase the plant growth, particularly under stress conditions, by modulating the enhanced ethylene production in response to a multitude of abiotic and biotic stresses including drought [136,137,138]. Therefore, PGPRs reduce adverse effects of so-called stress ethylene.

Wheat seedling primed with ACCd-containing strain *Bacillus subtilis* LDR2 showed a diminished content in ACC and a better photosynthetic efficiency under drought stress [45]. Similarly, priming of wheat seedlings with strain *Klebsiella* sp. IG 3 lead to higher root length and number, enhanced fresh and dry weight of shoots and roots and better RWC [31]. Nevertheless, in this study, the used PGPRs have several other PGPR traits such as production of ACCd but also production of IAA or EPS. However, globally, an inoculation with rhizobacteria containing ACC deaminase might be helpful in removing the inhibitory impacts of drought stress on plant growth.

### 3.7. Phytohormones-Dependent Drought Signal Pathways

Phytohormones play a crucial role in the normal development and growth of plants, but they also have an importance in stress response, including drought [52]. Some PGPRs are able to directly produce or to trigger the production of phytohormones by plants and, thus, affect hormonal balance within the plant. The bacterial production of phytohormones is mainly limited to auxins: indole-3-acetic acid (IAA), indole-3-carboxylic acid (ICA) or indole-3-lactic acid (ILA).

The production of auxins by PGPRs may modify the RSA [139]. It is of special interest in wheat, since it increases root surface area and, thus, allows a better assimilation of water and nutrients, improving global plant growth and health during drought stress [67]. Among auxins, the physiologically most active one is indole-3-acetic acid [52]. Inoculation of wheat seedlings with IAA-producing strain *Klebsiella* sp. IG 3 leads to improve root length and number during drought stress. It triggered higher fresh and dry weights of roots and shoots [31]. These changes in root morphology are associated with the drought tolerance improvement [63]. In response to water shortage, the wheat inoculated with strain *Azospirillum* sp. B3 also showed better root growth, as well as nutrient and water assimilation, partly due to the bacterial production of IAA [119]. Inoculation of auxin-producing rhizobacteria of genus *Bacillus*, *Enterobacter*, *Moraxella* and *Pseudomonas* lead to significant improvement of shoot length, spike length and grain weight in wheat under drought conditions at 10% field capacity. Combinations of several different strains lead to significant improved yield parameters [140]. Khalid et al. [122] established a significant linear correlation between the in vitro production of IAA by bacteria and their capacity to improve the wheat tolerance to drought stress. They also found, during pots and field trials, a positive correlation between the increase in yield and the root development for the selected bacteria [122]. Some PGPRs are also able to modify the plant IAA concentration through an indirect pathway. Wheat seedling primed with strain *B. subtilis* LDR2 showed an increase of IAA content by 80% and a better photosynthetic efficiency under drought stress, when compared to non-inoculated plants. The PGPR used was, thus, able to increase the plant IAA content through modulation of IAA synthesis and signaling pathway [45].

The content of abscisic acid may be modified in plants inoculated with PGPRs. Indeed, wheat seedling primed with strain *Bacillus subtilis* LDR2 showed a lower concentration of ABA during drought stress, compared to non-inoculated seedlings, likely due to the production of ACCd by the bacterium [45]. In fact, it has already been shown that ethylene has an impact on ABA biosynthesis [141]. In inoculated plants, the ABA content decreased by 30%, when compared to non-primed stressed control, while the shoot dry weight increased by 28% and the root dry weight by 17% [45].

## 4. Conclusions

**Figure 6:**
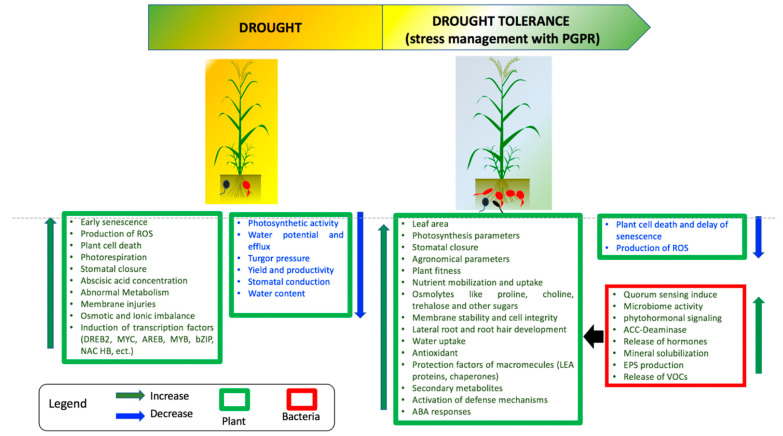
Events triggered in plant by the drought stress and mechanisms used by PGPR to alleviate this stress.

The present review summarizes the current knowledge on effects of the drought stress on wheat plants and the use of PGPRs as a solution for improving drought stress tolerance (Figure 6). During the water shortage on wheat, a wide variety of physiological, molecular and anatomical aspects are affected by drought stress, such as photosynthesis, growth, osmotic and oxidative status and even lipid membranes.

Several studies focused on effects of PGPR inoculation on wheat drought tolerance, because of the agronomic and economic stakes. Nowadays, it is well known that PGPRs may increase root and shoot growth and even yield of wheat plants through different modes of action. It includes the production of ACCd, EPS or phytohormones, but so far, much remains to be learned in regard to the exact mode of action. Indeed, a beneficial physiological effect observed on a plant can be due to one or several PGPRs using one or several of the mechanisms described above. It remains difficult to determine which mechanism is responsible for which effect in plant. Some mechanisms, such as modification of antioxidants or osmolytes concentration/activity, remain unclear. For example, when an increased concentration of antioxidants is measured in wheat, we do not know if this amount is due to the bacterial antioxidant production or if the bacteria induced plant antioxidant biosynthesis [2]. The link between the potential of the strain and the observed effects on plants is not always evident. The published analyses do not always study the mechanisms behind, but are often limited only to growth, biomass or yield measures. When mechanisms are studied, they often consist in only one analysis, which is not enough to understand the whole mechanism. Further, strains often possess several PGPR characters and studies with specific knock out mutants could be useful to confirm and quantify the contribution of the different mechanisms involved in the drought tolerance. Tools such as transcriptomics, proteomics or metabolomics may also be powerful to enhance the knowledge regarding the mechanisms involved [142]. In the interaction between wheat and PGPR, the main studied phytohormones is IAA, but impacts of other hormones, such as CK or BR, remain poorly studied. The improved drought tolerance of wheat by PGPR remains a promising and challenging solution, however their mode of action are not fully understood.
